# Supplementary material for: Transition metal dichalcogenides bilayer single crystals by reverse-flow chemical vapor epitaxy
Source: Nat Commun. 2019 Feb 5;10:598. doi: 10.1038/s41467-019-08468-8 (PMC6363754; doi:10.1038/s41467-019-08468-8)
Supplement: Supplementary file 1 — Supplementary Information [file 41467_2019_8468_MOESM1_ESM.pdf]

Supplementary Information

Transition metal dichalcogenides bilayer single crystals by reverse-flow chemical vapor epitaxy

Xiumei Zhang[1,2,#], Haiyan Nan[1,#], Shaoqing Xiao[1,*], Xi Wan[1], Xiaofeng Gu[1,*], Aijun Du[3], Zhenhua Ni[4] and Kostya (Ken) Ostrikov[3,5]

[1] *Engineering Research Center of IoT Technology Applications (Ministry of Education), Department of Electronic Engineering, Jiangnan University, Wuxi 214122, China.*

[2]*School of Science, Jiangnan University, Wuxi 214122, China.*

[3]*Institute for Future Environments and School of Chemistry, Physics and Mechanical Engineering, Queensland University of Technology, Brisbane QLD 4000, Australia.*

[4]*Department of Physics and Key Laboratory of MEMS of the Ministry of Education, Southeast University, Nanjing 211189, China.*

[5]*CSIRO-QUT Joint Sustainable Processes and Devices Laboratory, Commonwealth Scientific and Industrial Research Organization, P.O. Box 218, Lindfield NSW 2070, Australia.*

[#]*These authors contributed equally to this work.*

*Correspondence and requests for materials should be addressed to Shaoqing Xiao (email: larring0078@hotmail.com) or to Xiaofeng Gu (email: xgu@jiangnan.edu.cn).

Supplementary Note I. Challenges in TMD bilayer growth.

The temperature program of the modified sequential two-stage thermal CVD process is presented in Supplementary Figure 1. The layer-by-layer growth of 2D TMD materials is challenging due to extremely delicate nature of the atomically thin crystals and the highly sensitive growth conditions. TMD monolayer like MoS_2 monolayer crystals were often achieved in conventional CVD synthesis with constant working temperature. Indeed, we did obtained triangle MoS_2 monolayer crystals (Supplementary Figure 2) in the modified thermal CVD system when the growing temperature was maintained at 700 throughout the whole growing process from A-B to C-D stage. This could be well understood since the laterally epitaxial growth dominated such growing process. It is clear that the optical contrast (Supplementary Figure 2a) between the MoS_2 monolayer and the substrate is lighter than that between the MoS_2 bilayer and the substrate (Figure 1b). The AFM surface morphology as well as the height profile reveals a thickness of 0.78 nm, matching well with the previous reports on CVD-grown MoS_2 monolayer crystals[1, 2].

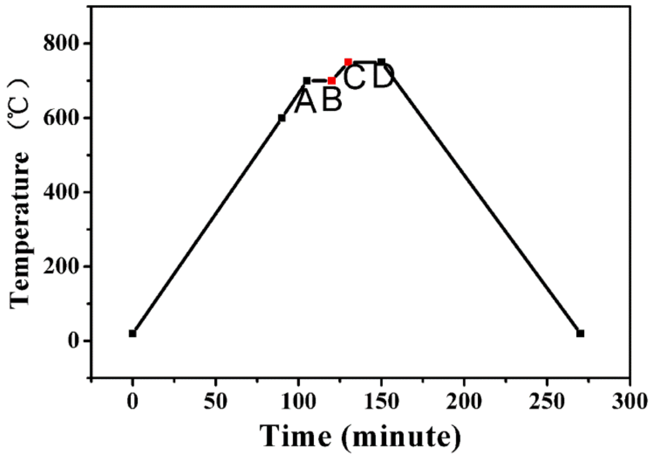

Supplementary Figure 1. The temperature program of the modified sequential two-stage thermal CVD process: A-B stage stands for the growth of first layer, C-D stage represents the growth of second layer, while B-C stage corresponds to the growth swing stage for the first and second layer.

Therefore, the layer-by-layer growth of TMD materials requires incrementally higher growing temperatures[3-6]. To grow TMD bilayer crystals, we intentionally divided the sequential growth process into two stages with different growing temperatures: one (A-B

stage) is relatively lower temperature for the growth of first monolayer and the other (C-D stage) is relatively higher temperature for the vertically epitaxial growth of second monolayer. However, in such a typical sequential CVD growth process with the forward gas carrier flow direction, the uncontrolled nucleation during the temperature rising process (B-C stage) between these two sequential growth stages represents the main obstacles to reliable formation of TMD bilayer crystals. Indeed, the uncontrolled nucleation can result in many triangle monolayer crystals (second layer) randomly growing on the surface of the first monolayer crystals, as shown in Supplementary Figure 3.

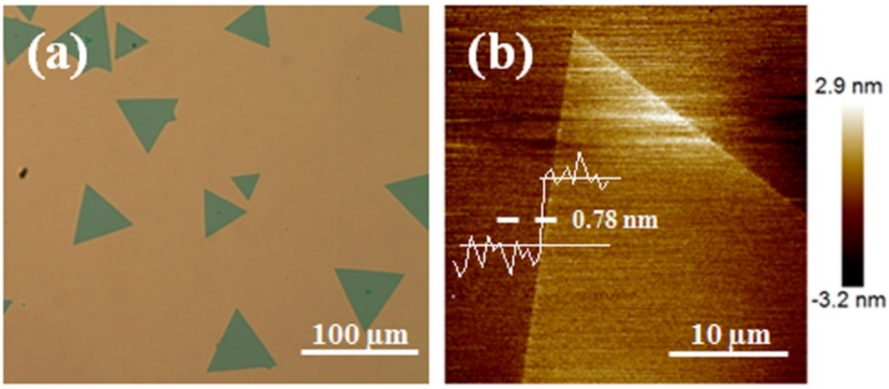

Supplementary Figure 2. **a** The representative optical image and **b** AFM surface morphology of the as-grown MoS_2 monolayer crystals by the modified CVD with constant working temperature at 700 □. Inset in **b** shows the height profile across the monolayer surface and the substrate surface.

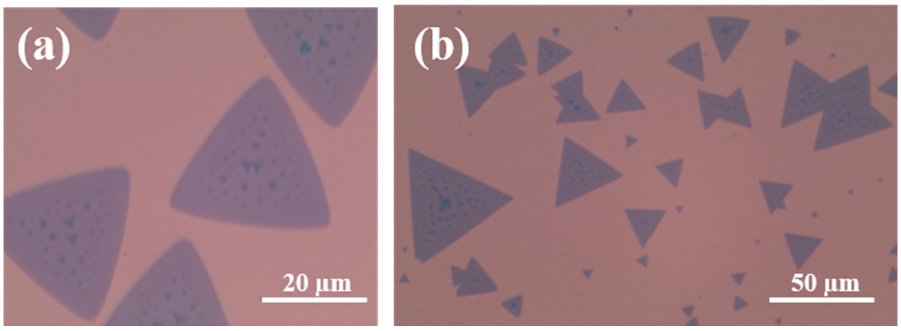

Supplementary Figure 3. **a-b** The optical images of the MoS_2 bilayer crystals with many small triangle monolayer crystals (second layer) randomly distributed on the surface of the first monolayer crystals due to the uncontrolled nucleation during the temperature rising process.

To solve this problem, we introduced a reverse N_2/H_2 flow from the substrate to the source during the temperature rising stage (B-C stage). The reverse flow could not only saturate the dangling bonds on the edge and at the surface of the first MoS_2 monolayer but also prevent unintended supply of chemical vapor source to eliminate uncontrolled nucleation. As such, the growth of MoS_2 bilayer crystals can be significantly promoted, as shown in Figure 1b. Furthermore, some adjacent MoS_2 bilayer domains could meet each other and merger together to form a continuous bilayer film as shown in the representative optical images and SEM surface image (Supplementary Figure 4). In some area, one can clearly observe the grain boundaries between the adjacent bilayer domains.

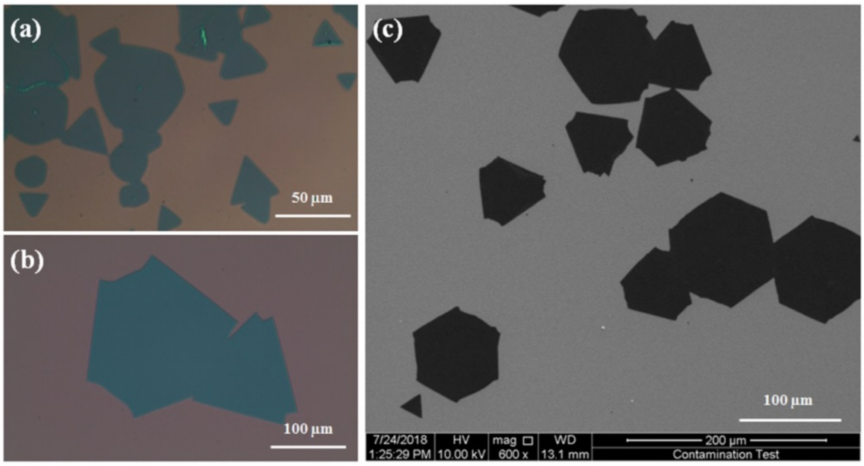

Supplementary Figure 4. **a-b** The representative optical images and **c** SEM image of continuous MoS_2 bilayer films consisting of adjacent MoS_2 bilayer domains.

From these bilayer images, we can observe that triangular and hexagonal shapes dominate over the resultant bilayer domains. Based on the principles of crystal growth, the shape of a crystal is determined by the growth rate of different crystal faces. The fastest growing faces either become smaller or disappear while the slowest growing faces become the largest. For CVD grown MoS_2, the most commonly observed growing faces are Mo zigzag (Mo-zz) terminations and S zigzag (S-zz) terminations, which can be influenced by the ratio of Mo and S atoms on the growing substrate[3]. In detail, in a Mo sufficient atmosphere, S-zz terminations grow faster than the Mo-zz terminations, so the domain shape will be a

triangle with three sides of Mo-zz terminations. When the Mo:S ratio is close to the stoichiometric ratio of MoS_2 (1:2), the termination stability and the probability of meeting free atoms are similar for both Mo-zz and S-zz terminations, which results in similar growing rates. In this case, the final shape of the domains will be hexagon. In a S sufficient atmosphere, the domain shape will also transform to triangular with three sides of S-zz terminations. Furthermore, the ratio of Mo and S atoms on the growth substrate may vary along the gas flow direction, leading to the coexistence of different shapes.

In theory, Cao et al.[7] revealed that the chemical potential played a crucial role in the determination of equilibrium shape of MoS_2 using DFT calculations and Wulff construction rule. In a S-rich condition, the S chemical potential is high and the Mo edge with S termination (named zz-S2) is the most stable edge structure because its formation energy is lowest. On the basis of crystal growth theory, the low-energy edges will preserve and active edges will disappear during the growth. Therefore, such a S-rich condition will lead to a triangular equilibrium shape of MoS_2 in the end. When the concentration of S is reduced in the growth process, the S chemical potential will decrease. In this situation, the zz-S and zz-S2 edges will be enlarged, finally leading to a hexagonal shape of MoS_2. Schweiger et al.[8] have also reached the same conclusion that a high chemical potential of S may result in triangular-shaped particles terminated by the Mo-edge terminated surface based on DFT calculations and Gibbs-Curie-Wulff equilibrium morphologies.

In our case, we used 15 mg MoO_3 powder and 100 mg S powder, which corresponded to a S-rich atmosphere. Therefore, the resultant domains are dominantly triangle as observed. However, as the reaction continued, the S concentration would reduce and vary along the gas flow direction especially on the growing surface inevitably, so the S chemical potential would decrease and the Mo:S ratio on the growth substrate would even be close to 1:2. In such case, hexagon domain shapes would be obtained. Indeed, triangular and hexagonal MoS_2 domains can coexist on the growth substrate as observed.

The different growing temperature at C-D stage can result in bilayer MoS_2 crystals with different stacking structures. Supplementary Figure 5 presents the evolution of the optical images of the as-grown samples with the growing temperatures of C-D stage for the second layer growth. For both 700 and 720 ℃, one can observe MoS_2 monolayer crystals

dominating over the resultant flakes on the substrate surface no matter how we prolong the growth time of C-D stage. For both 750 and 770 ℃, uniform AA stacking MoS_2 bilayer crystals are largely distributed on the substrate surface. For 790 ℃, however, both AA and AB stacking MoS_2 bilayer crystals coexist on the same substrate surface. For 800 ℃, AB stacking MoS_2 bilayer crystals become dominant over the resultant flakes on the substrate surface.

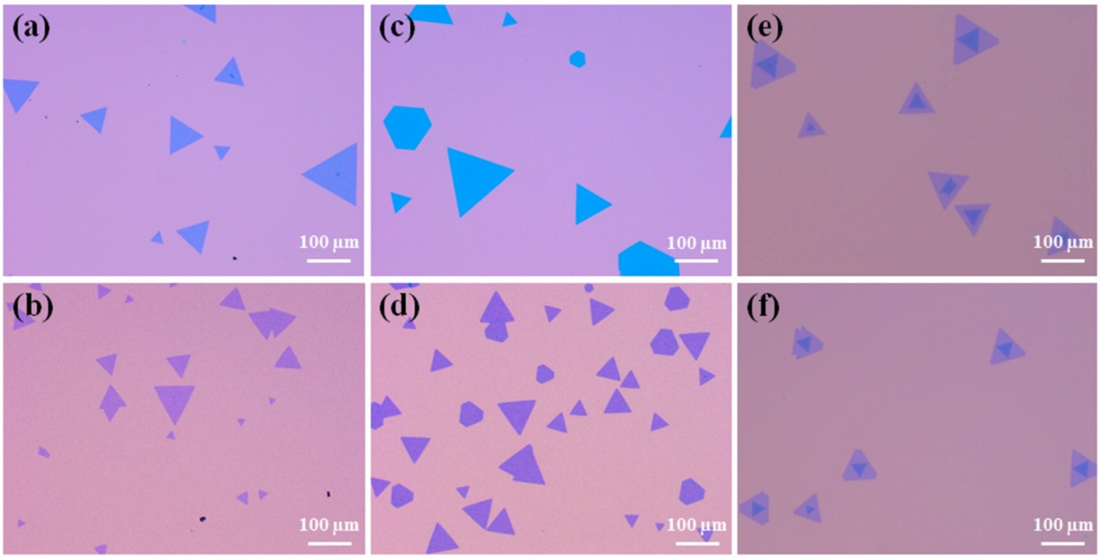

Supplementary Figure 5. The representative optical images of MoS_2 crystals obtained under different growing temperatures of C-D stage: **a** 700 ℃, **b** 720 ℃, **c** 750 ℃, **d** 770 ℃, **e** 790 ℃ and **f** 800 ℃.

As we emphasized above, the reverse flow can prevent unintended supply of chemical vapor source to eliminate the generation of new nucleation centers on the growth substrate and the as-grown first layer and thus promote the epitaxial growth of second monolayer, but is not the only deciding factor of the different stacking orders. In fact, the AA and AB stacking orders are decided by their stability and thermodynamic energy. The stability of the bilayer crystals is related to the formation energies, defined as the total energy difference per atom between bilayer and the two constituent but separated single monolayers ($E_{form\text{-}2L} = E_{bilayer}\text{-}2E_{monolayer}$). The larger the absolute value of the formation energies, the more stable the growing structure. Therefore, the growth of every monolayer is sensitive to the growth parameters, especially the growth temperature difference between the two adjacent

monolayers and the gas flow rate during the growing process.

To our best knowledge, there had already been several theoretical calculations on bilayer MoS_2 with at least five different stacking patterns before bilayer MoS_2 was synthesized in experiment. All these reports reached the same conclusion that AA stacking with the twist angle of 0^0 (corresponding to 3R like phase) and AB stacking with the twist angle of 60^0 (corresponding to 2H phase) are the most energetically stable bilayer crystals[3,4,9,10]. This is because the alignments of Mo and S atoms between different layers under such two stacking conditions can make the energy of the system smaller than those under other stacking conditions. With the development of experimental synthesis technology in recent years, bilayer MoS_2 crystals have been successfully synthesized by CVD and these bilayer crystals typically exhibit only two stacking patterns namely AA and AB stacking corresponding to 3R like and 2H phase, respectively[3,4,9]. All these reported experimental results collectively suggest that AA and AB stacking patterns are the most thermodynamically stable ones. In a word, AA (3R like phase) and AB (2H phase) stacking phases are the most energetically stable and commonly observed phases in natural and synthetic MoS_2 bilayer crystals[9].

The growing results under different temperature (e.g., 700 ℃, 720 ℃, 750 ℃, 770 ℃, 790 ℃ and 800 ℃) of C-D stage suggest that the growth temperature difference between the two adjacent monolayers together with the reverse flow determines the stacking order of such bilayer growth. It has ever been reported that bare SiO_2/Si is more wettable and has higher total surface energy when compared to MoS_2/SiO_2/Si[11]. In addition, the increasing growing temperature can reduce the wettability of the growth substrate[11]. For 720 ℃ or even lower, the increasing growing temperature between A-B stage and C-D stage is not large enough to reach a low enough wettability so that vertical epitaxy is suppressed leading to monolayer crystals dominating over the resultant flakes. For 750 ℃ or even higher, the increasing growing temperature is large enough which can effectively suppress the new nucleation on the substrate and lead to the vertically epitaxial growth on the as-grown monolayer MoS_2[12]. For 750~790 ℃, the growth temperature difference between the two adjacent monolayers is suitable for the formation of AA stacking. For 800 ℃ or even higher, the growth temperature difference between the two adjacent monolayers is optimum for the formation of AB stacking. This is quite consistent with the theoretical prediction that AB (2H

phase) stacking may become progressively more stable with increasing temperature[13].

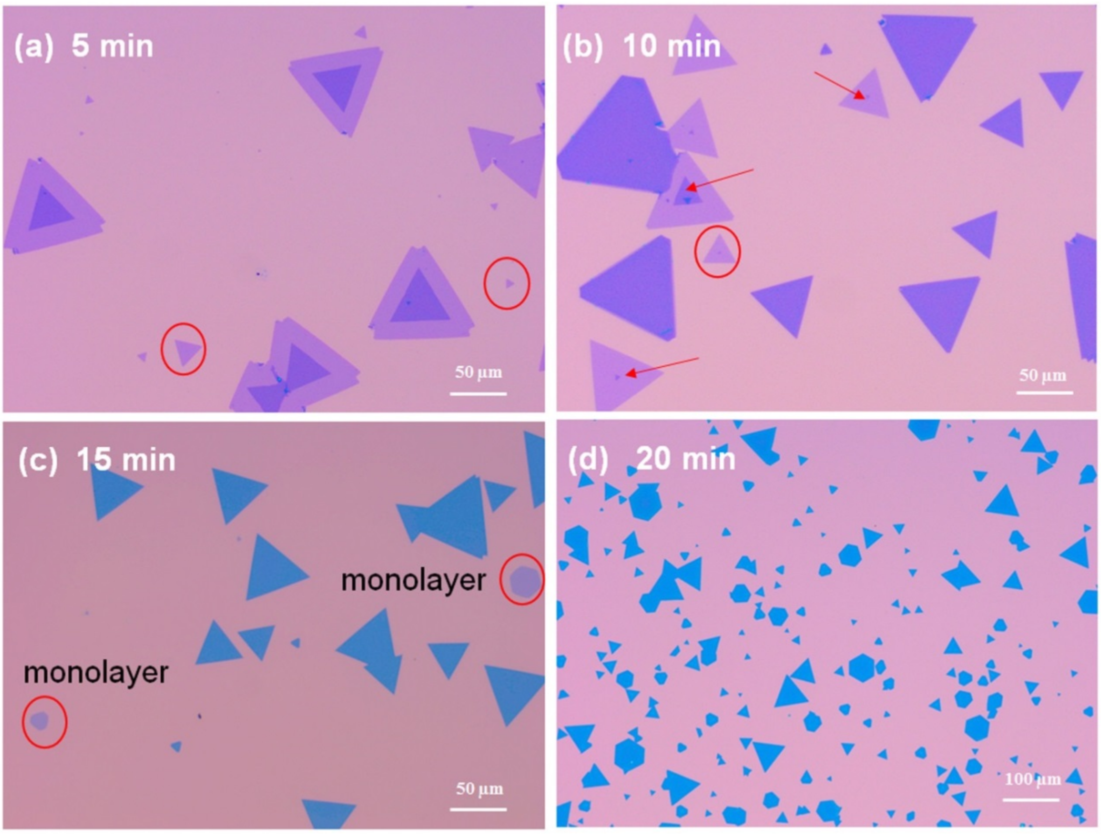

Supplementary Figure 6. The representative optical images of AA stacking MoS_2 bilayer samples obtained under different growth time of C-D stage at 750 ℃ (for the second layer growth): **a** 5 min, **b** 10 min, **c** 15 min, and **d** 20 min.

The structure of the as-grown AA stacking bilayer MoS_2 samples displays a close relationship with the growth time of C-D stage (for the second layer growth). However, it is hard to quantitatively study the size of the second layer MoS_2 with the growth time for the flowing reasons. First, the size of the second layer is bound by the first layer, in other words, the edge of the first layer acts as a limiting factor in the growth of the second layer. It is difficult to overcome the potential barrier of the steps between the first layer and the growth substrate. Second, the size of the monolayer MoS_2 grown at stage A-B usually varies from sample to sample leading to the different size distribution of the resultant bilayer crystals. Third, it couldn't be confirmed that all the second layers start the growth on the as-grown monolayers simultaneously so that the size of the second layer can change from flake to flake

even on a same growing substrate. Even so, we can qualitatively study the size of the second layer MoS_2 with the growth time at 750 ℃ and the results are shown in Supplementary Figure 6. The overall trend is the transition from incomplete AA stacking with steps to complete AA stacking with the increase in the growth time of C-D stage for the second layer growth.

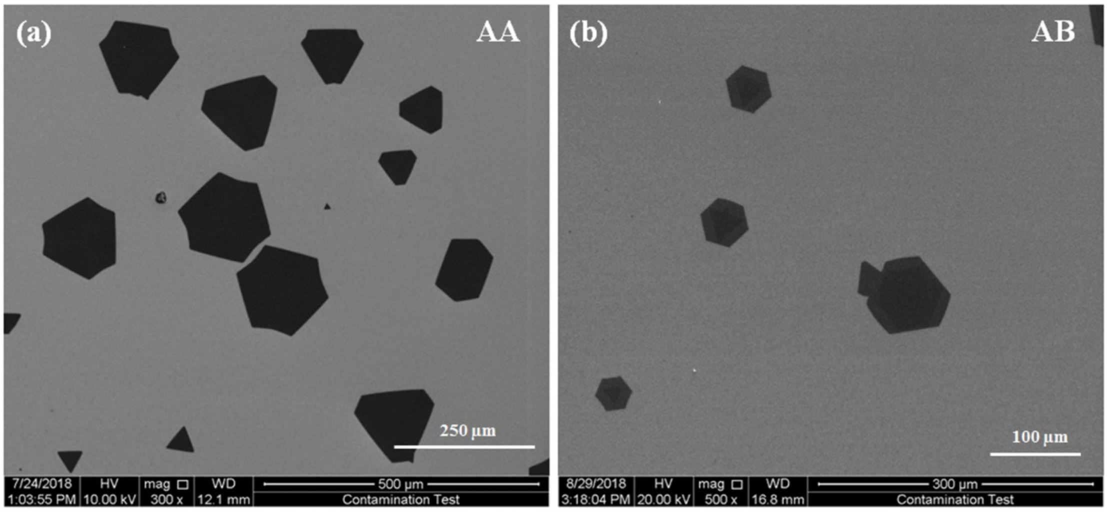

Supplementary Figure 7. The representative SEM image of the as-grown MoS_2 bilayer crystals with **a** AA and **b** AB stacking orders.

Supplementary Figure 7 shows the representative SEM images of the as-grown MoS_2 bilayer crystals with AA and AB stacking orders. For AA stacking ones, the thickness are very uniform. For AB stacking ones, one can clearly distinguish between the monolayer region and the bilayer region by the different color depth.

Supplementary Note II. TMD trilayer and multilayer growth

Taking the reverse flow epitaxy method a step further, we can also grew MoS_2 trilayers including AAA, AAB, ABB as well as ABA stacking orders and even multilayers as shown in Supplementary Figure 8. By setting reasonable temperature steps and gas flow rates for the third layer growth, we obtained large-area and large-size AAA and AAB stacking trilayer crystals as clearly reflected by the optical images (Supplementary Figures 8a-8b and 8d) and

the AFM surface morphology picture (Supplementary Figure 8c). However, those MoS_2 trilayer crystals with other stacking orders like ABB and ABA can be occasionally obtained and usually coexist on the same substrate. The formation and coexistence of different stacking MoS_2 trilayer crystals may be related to their formation energies and their stability. Similar to the case of bilayer crystals, the formation energies of trilayer crystals can be defined as the total energy difference per atom between the trilayer and both the bilayer and monolayer ($E_{form-3L} = E_{trilayer} - E_{monolayer} - E_{bilayer}$). The growth parameters (including the selectivity between the AAA and AAB stacks as well as between the ABA and ABB stacks) at this stage are not optimized and it will be the subject of future work. For the multilayer growth, the growing temperature of C-D stage was increased gradually for first 10 minutes and then kept constant for subsequent 10 minutes other than being constant throughout the growing stage.

The experimental results on trilayer crystals (Supplementary Figure 8a-f) indicate that the four stacking orders namely AAA, AAB, ABA and ABB are more energetic favourable. This could be easily understood because the layer by layer growth is universal for bilayer, trilayer and even multilayers. MoS_2 is interacted by weak van der Waals force between layers and between the bottom layer and the substrate. For trilayer, we can treat the bottom layer as the substrate and the second layer as the first layer as displayed in the previous report[14]. As such, the stacking order of the third layer with the second layer can be the same as bilayer models. When the bottom bilayer MoS_2 is with AA stacking order, the third layer can has a twist angle of 0° or 60° with the second layer, which can be labelled as AAA or AAB stacking following the nomenclature of bilayer system. When the bottom bilayer MoS_2 is with AB stacking order, the third layer can also has a twist angle of 0° or 60° with the second layer, which can be labelled as ABA or ABB stacking. The trilayer MoS_2 crystals with such four stacking patterns including AAA, AAB, ABA and ABB have indeed been synthesized by our reverse-flow chemical vapour epitaxy method (Supplementary Figure 8a-f), proving that they are energetically favourable configurations, and this is quite consistent with the previous report[3].

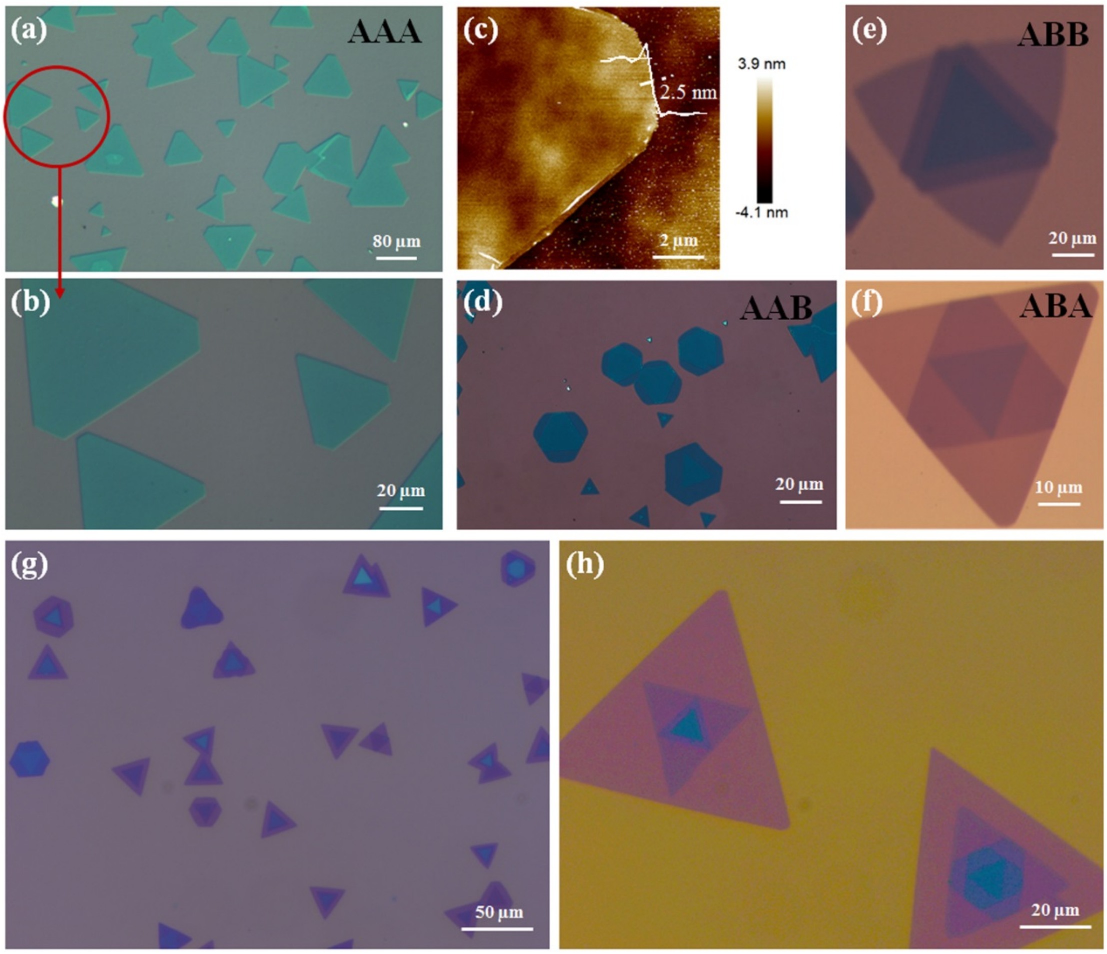

Supplementary Figure 8. **a-b** The optical images of trilayer MoS_2 with AAA stacking order; **c** the AFM surface morphologies of the trilayer MoS_2 with AAA stacking order; the optical images of the trilayer MoS_2 with **d** AAB, **e** ABB and **f** ABA stacking order; **g-h** the optical images of multilayer MoS_2.

In combination with the experimental results on multilayer crystals (Supplementary Figure 8g and 8h), we can reach a conclusion that every upper layer can has a twist angle of 0° or 60° with its neighborhood bottom layer for every energetically favourable MoS_2 crystals including bilayer, trilayer, four-layer and even muitlayers. Yan et al.[3] calculated the adhesion energies of four-layer MoS_2 in theory to understand their growth behaviors. They started with a monolayer MoS_2, then added one more layer each time and explored all the possible configurations. They gave the following remarks: at the initial stage of MoS_2 CVD growth, MoS_2 can adopt various stacking configurations due to the comparable adhesion energies. This is the reason why we can obtain a lot of trilayer and even multilayer MoS_2 crystals with various stacking patterns on the same growth substrate under certain conditions.

Supplementary Note III. PL and Raman characterization of the as-grown MoS_2 bilayer crystals

Apart from the mapping studies on the complete AA stacking and AB stacking samples, we also performed Raman and PL mapping measurements on the incomplete AA stacking domains with distinct steps and the results are shown in Supplementary Figure 9. As seen, there are clear differences between the monolayer and the bilayer area. The Raman intensity of E_{2g}^1 mode of the bilayer MoS_2 in the centre area is much stronger than that of the monolayer one on the edge, while the PL intensity of the monolayer MoS_2 on the edge is evidently much stronger than that of the bilayer one in the centre area.

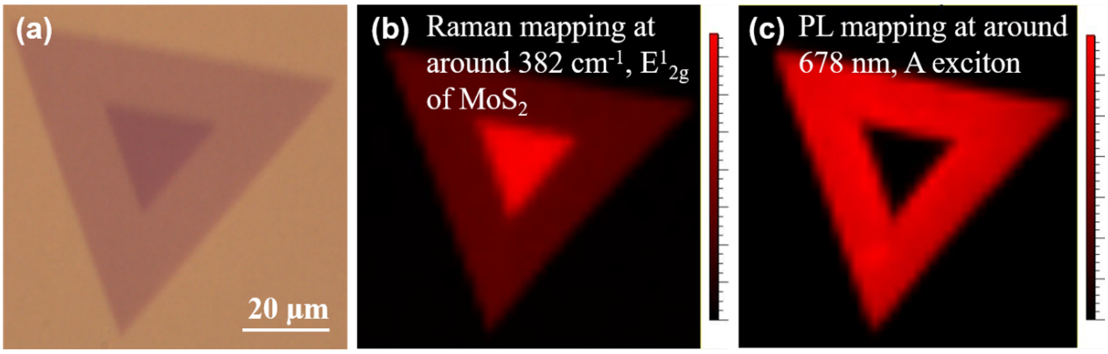

Supplementary Figure 9. **a** optical image, **b** Raman intensity mapping at around 382 cm^{-1} (E^1_{2g} mode) and **c** PL intensity mapping at around 678 nm (A exciton) of an incomplete AA stacking bilayer MoS_2 sample with steps between the two layers.

The structural characteristics of MoS_2 bilayer crystals are indeed not sensitive to the flake size. It has been proved that the uniformity of the layer number can be identified by the optical contrast between the flakes and the substrates[15]. The uniform optical contrasts of the optical images shown in this work strongly prove the uniformity of the as-grown bilayer flakes. Here we also performed Raman and PL mapping studies on two small MoS_2 bilayer flakes (AA and AB stacking) with a size of about 20 μm in Supplementary Figure 10 and 11, respectively. The highly uniform distribution of both Raman intensity (Supplementary Figure 10a-b) and PL intensity for the three dominant peaks A, B and C (Supplementary Figure 11a-c) across the whole domain reflect the high homogeneity of the as-grown AA stacking domain. For the as-grown AB stacking MoS_2 bilayer domain, one can clearly observe the boundary

between monolayer region and bilayer region from both Raman intensity mappings of E_{2g}^{1} and A_{1g} (Supplementary Figure 10c-d) as well as PL intensity mappings of peak A, B and C (Supplementary Figure 11d-f). This can be easily understood because of the fact that the Raman signal is stronger on the bilayer region than on the monolayer region, and that the PL signal of peak A is stronger on the monolayer region than on the bilayer region while the PL signals of peak B and C only emerge on the bilayer region. The results reflect the same properties of the small flakes as those of the big flakes (Figure 3). Nevertheless, both Raman and PL intensity on the boundaries of the big flakes are much clearer than those of the small flakes.

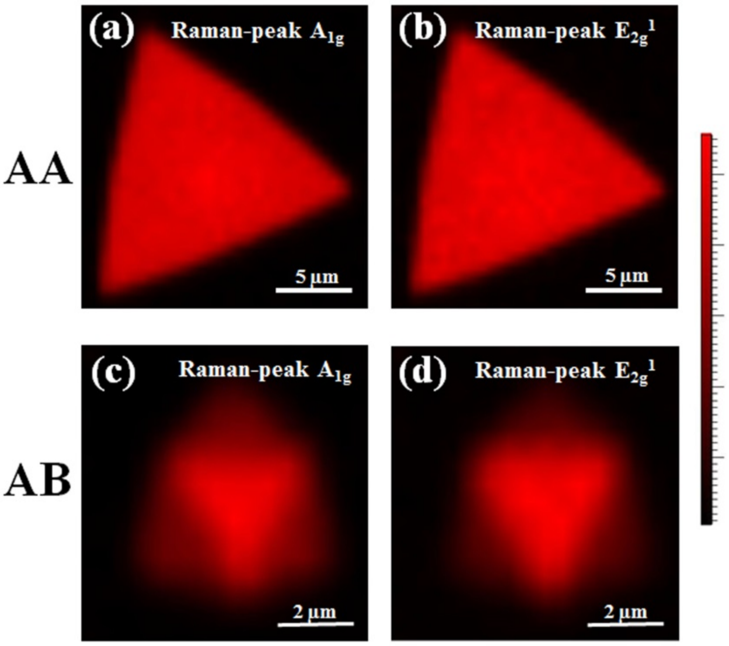

Supplementary Figure 10. Raman intensity maps of two small MoS_2 flakes with a size of about 20 µm: **a-b** AA stacking order and **c-d** AB stacking order.

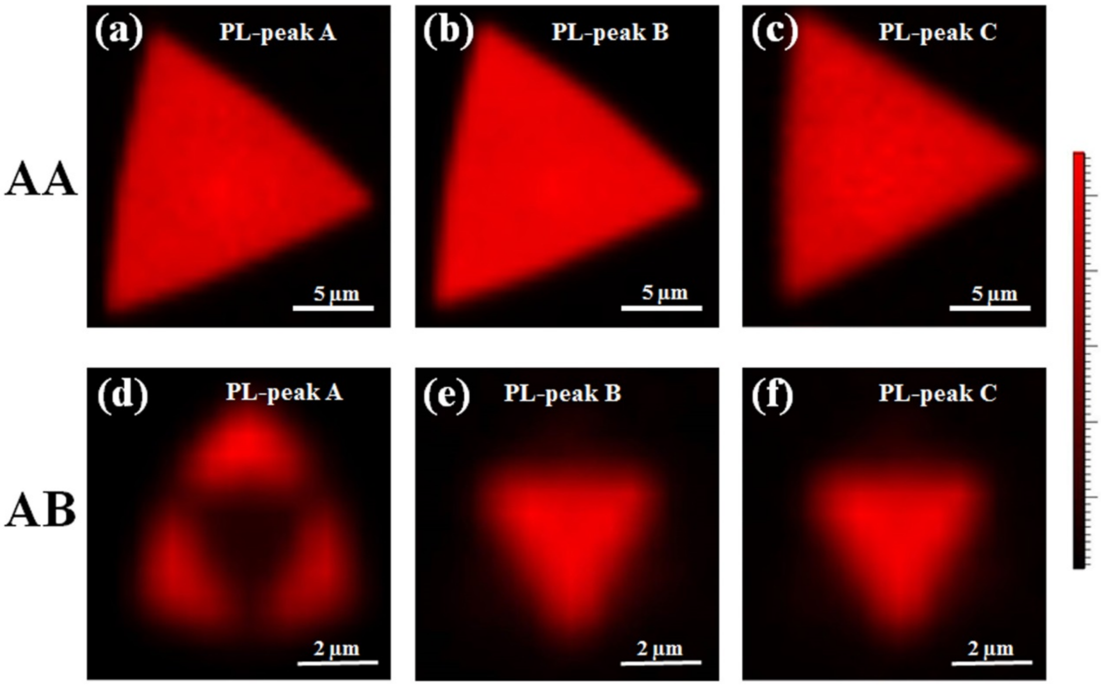

Supplementary Figure 11. PL intensity maps of two small MoS_2 flakes with a size of about 20 μm: **a-c** AA stacking order and **d-f** AB stacking order.

Supplementary Note IV. DFT-D2 dispersion correction method for van der Waals interaction

In this work, we adopt the Grimme's DFT-D2 dispersion correction approach because this method can be applicable for all exchange correlation energies due to its higher accuracy and less empiricism[16]. In a recent paper[17], the authors compared all the van der Waals methods and demonstrated that DFT-D2 method can work very well for layered materials. Furthermore, such approach has been widely adopted in the calculation of van der Waals interaction system such as transition-metal dichalcogenides (TMDs)[18] and could yield very good interlay distances[13], which are proved to have significant effect on the band structures[19].

In order to prove the validity of this method, we also benchmark our calculated results of monolayer MoS_2 with other available publications in terms of lattice constant, bond distance and bond angle. The calculated lattice constant of monolayer MoS_2 is 3.16 Å, which is in good agreement with the previous calculation and experiment results[20,21]. The optimized bond distance between Mo and S atoms is 2.41 Å, and the angle between Mo–S bonds is 81.78°, both of which agree well with the previously reported theoretical calculation results[22]. The

calculated results of monolayer MoS_2 show that a direct bandgap of 1.79 eV is located at the K point, matching well with the experimental data[23].

We also performed theoretical calculations using DFT-D2 correction method on all five possible high-symmetry stacking orders: (a) AB' (point group D_{3h}): eclipsed stacking with Mo over Mo and S over S; (b) AB (point group D_{3d}): eclipsed stacking with Mo over S, characteristic of the 2H phase; (c) A'B (point group D_{3d}): staggered stacking with S over S; (d) AA (point group C_{3v}): staggered stacking with S over Mo, characteristic of the 3R phase; (e) AA' (point group D_{3d}): staggered stacking with Mo over Mo. The corresponding side and top views of the five stacking orders are shown in Supplementary Figure 12. It is clear that one can transform one stacking poly-typism into another by horizontal layer sliding and/or by rotation around the vertical axis. Supplementary Table 1 provides the detailed comparison in terms of lattice constant (A), Mo-S bond length (L), S-Mo-S bond angle (θ) and total energy (E_{total}) between the five stacking orders. The corresponding bulk experimental values[24] are used as a reference.

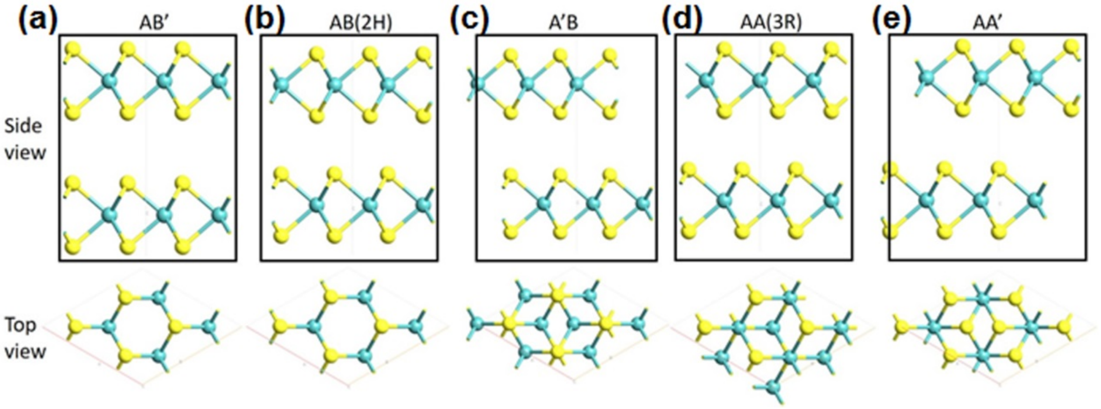

Supplementary Figure 12. Side view and top views of the five possible high-symmetry stacking orders: **a** AB' (point group D3h): eclipsed stacking with Mo over Mo and S over S; **b** AB (point group D3d): eclipsed stacking with Mo over S, characteristic of the 2H phase; **c** A'B (point group D3d): staggered stacking with S over S; **d** AA (point group C3v): staggered stacking with S over Mo, characteristic of the 3R phase; **e** AA' (point group D3d): staggered stacking with Mo over Mo.

As seen, the lattice parameters including lattice constant, bond length and bond angle

match well with the experimental ones for all the five stacking orders. Of all the five stacking orders, AB stacking possesses the smallest system energy (E_{total}=-4830.6388 eV), which is only 1.6 meV lower than that (E_{total}=-4830.6372 eV) of AA stacking, while the other three stacking orders AA', AB' and A'B have much larger system energy with 179, 558 and 5618 meV higher than that of AB stacking, respectively, indicating that AB stacking is the most energetic favourable structure and next is AA stacking. The extremely small difference of system energy between AA and AB stacking is consistent with the previous calculation results[3,10] and can explain the occurrence of these two stacking orders in the natural bulk forms and CVD-grown samples[3,4,9].

Supplementary Table 1. Detailed Comparison in terms of lattice constant (A), Mo-S bond length (L), S-Mo-S bond angle (θ) and total energy (E_{total}) for the five stacking orders: AB', AB, A'B, AA and AA'. The corresponding bulk experimental values[24] are used as a reference.

Stacking	A (Å)	L (Å)	θ (^{O})	E_{Total} (eV)
AB'	3.161	2.41	81.76	-4830.5830
AB	3.161	2.41	81.78	-4830.6388
A'B	3.161	2.41	81.75	-4830.0770
AA	3.161	2.41	81.75	-4830.6372
AA'	3.161	2.41	81.77	-4830.6209
Bulk (Expt.)	3.160	2.42	82.00	

Supplementary Note V. Electrical performances of the as-grown MoS_2 bilayer based FETs

Supplementary Figure 13 displays the electrical performances including transfer characteristics (I_{DS}-V_g) and output characteristics (I_{DS}-V_{DS}) of the other three AA stacking bilayer MoS_2 FET devices: (a)-(b) AA 2; (c)-(d) AA 3; (e)-(f) AA 4. The calculated field-effect mobility values are 74.8, 43.6, 48.6 and 37.8 $cm^2V^{-1}s^{-1}$, respectively, for the four devices. The on/off ratios are estimated to be 3.6×10^4, 1.8×10^4, 1.0×10^4 and 1.0×10^4, respectively, for the four devices. The output characteristics (I_{ds}-V_{ds}) of all AA stacking devices are more likely to show linear and symmetrical relationships.

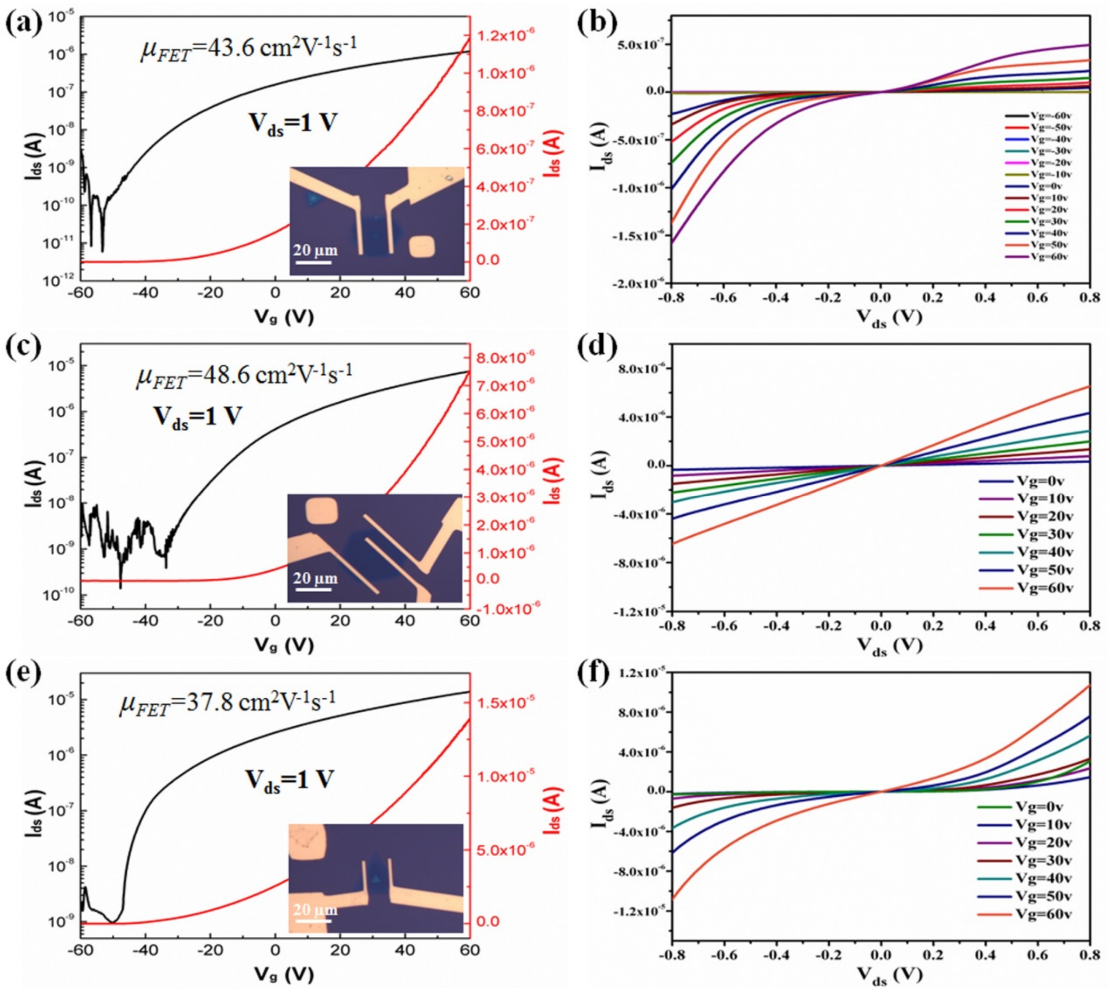

Supplementary Figure 13. The electrical performances including transfer characteristics (I_{DS}-V_g) and output characteristics (I_{DS}-V_{DS}) of the other three AA stacking bilayer MoS_2 FET devices: **a-b** AA 2; **c-d** AA 3; **e-f** AA 4. Insets in (a), (c) and (e) are the optical images of the corresponding FET devices.

Supplementary Figure 14 shows the electrical performances including transfer characteristics (I_{DS}-V_g) and output characteristics (I_{DS}-V_{DS}) of the other three AB stacking bilayer MoS_2 FET devices: (a)-(b) AB 2; (c)-(d) AB 3; (e)-(f) AB 4. The calculated field-effect mobility values are 7.4, 7.8, 4.3 and 1.0 $cm^2V^{-1}s^{-1}$, respectively, for the four devices. The on/off ratios are estimated to be 1.2×10^4,6.4×10^4, 6.2×10^4 and 4×10^3, respectively, for the four devices. The output characteristics (I_{ds}-V_{ds}) of all AA stacking devices show nonlinear and asymmetrical behaviors.

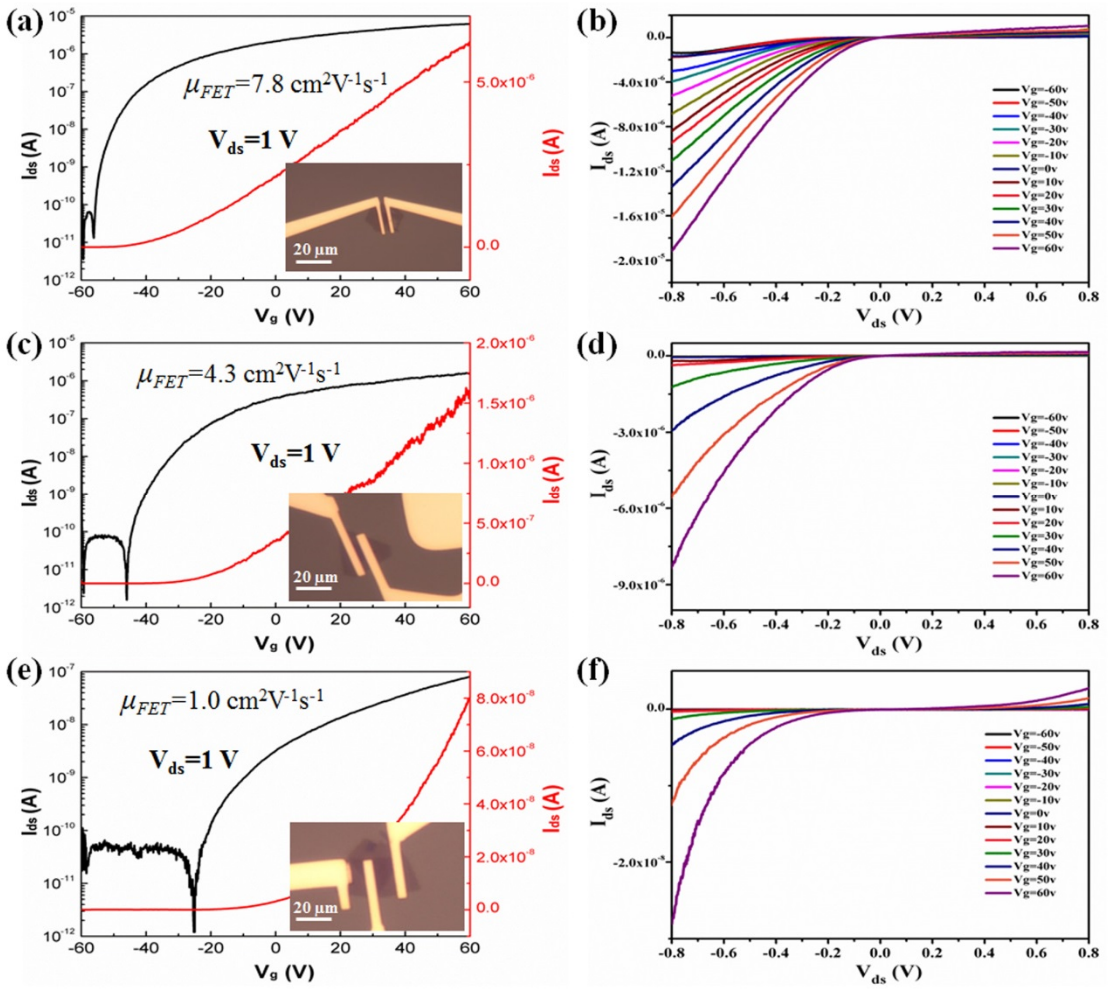

Supplementary Figure 14. The electrical performances including transfer characteristics (I_{DS}-V_g) and output characteristics (I_{DS}-V_{DS}) of the other three AB stacking bilayer MoS_2 FET devices: **a-b** AB 2; **c-d** AB 3; **e-f** AB 4. Insets in **a**, **c** and **e** are the optical images of the corresponding FET devices.

We have also fabricated FETs on the as-grown monolayer MoS_2 triangles by conventional CVD method for comparison and presented a set of representative results in Supplementary Figure 15. The output curves (I_{DS}-V_{DS}) show an extremely slight rectification characteristic, indicating the extremely slight Schottky contact between the electrode and monolayer. Similar results have also been reported previously[25]. In fact, whether for monolayer or bilayer MoS_2 FETs, the contacts can be of Ohmic and Schottky types as proved by many literatures[25-27]. The results varied from devices to devices. Just take the case of AA stacking bilayer MoS_2 FETs in this work, some are of Ohmic type as shown in Figure 5b and

Supplementary Figure 13d, and some are of extremely slight Schottky type as shown in Supplementary Figures 12b and 13f. However, for AB stacking ones in this work, all are of Schottky type. There are a lot of factors such as the work function of the electrode material, the layer number of MoS_2 and the unavoidable defects formed during the fabrication process that can influence the output characteristic[28,29].

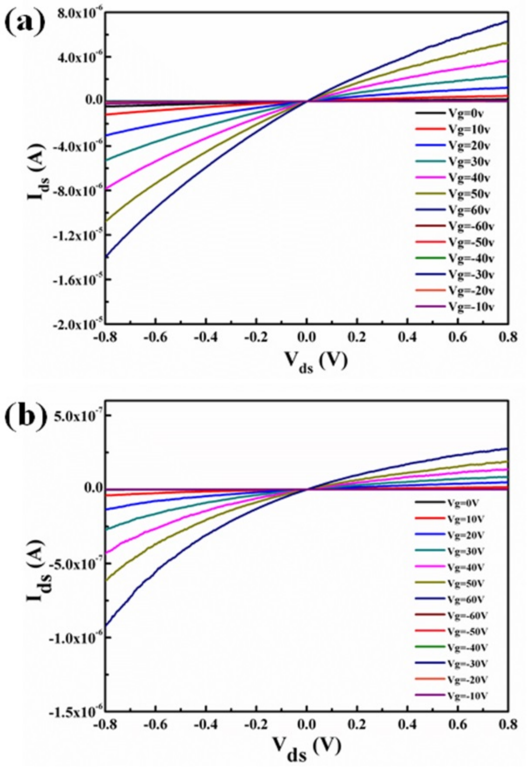

Supplementary Figure 15. The output characteristics (I_{ds}-V_{ds}) at varying Vg of two FETs based on conventional CVD-grown monolayer MoS_2.

Considering that the FET devices on both AA and AB stacking samples were fabricated with the same electrode materials and the same electron beam lithography (EBL) methods, the differences of output characteristic (I_{ds}-V_{ds}) between these two kinds of devices can only be related with their different stacking orders and their different contact arrangements/junctions with the metal electrodes. As shown in Supplementary Figure 16a, both the drain and source electrodes fall on the bilayer area for complete AA stacking bilayer FETs, so uniform bilayer acts as the FET channel. This situation is quite similar to MoS_2

monolayer based FETs. MoS_2 bilayer FETs have more electrons injected into the channel than the monolayer based FETs[30], leading to Ohmic contacts as reflected by the linear I_{ds}-V_{ds} plots shown in Figure 5b as well as Supplementary Figure 13. Similar results with Ohmic contacts between metal electrodes and MoS_2 bilayer have also been reported previously[31]. In contrast, there are steps between the two layers for AB stacking bilayer FETs, so the drain and source electrodes fall partly on the bilayer area and partly on the monolayer area as shown in Supplementary Figure 16b. Therefore, the electron density in the AB stacking bilayer channel are much lower compared to the AA stacking channel[30]. Furthermore, the stacking orders have also been shown to significantly affect the electronic characteristics of 2D materials[13,32-34]. Above all, we predict that the AB stacking order together with the uneven electrode distribution contribute to the Schottky contacts as revealed by the I_{ds}-V_{ds} curves in Figure 5d as well as Supplementary Figures 14b, 14d and 14f.

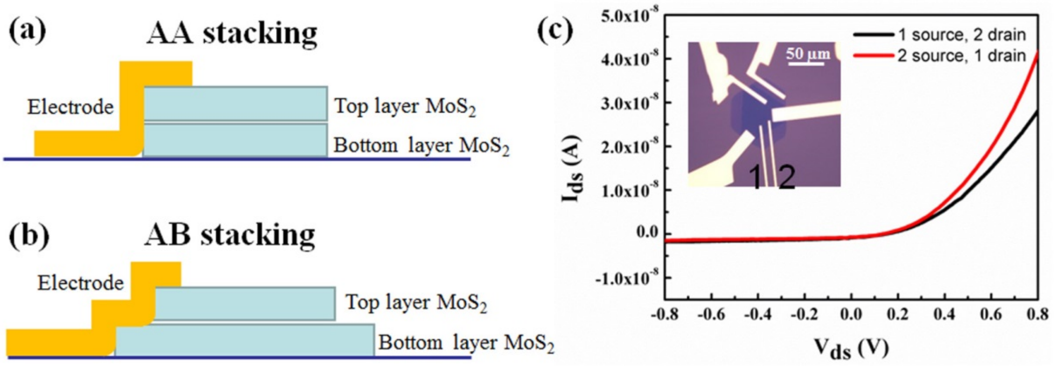

Supplementary Figure 16. The electrode configuration schematics of **a** AA and **b** AB stacking bilayer MoS_2 FET devices; **c** The output characteristics (I_{ds}-V_{ds}) of a representative AB stacking bilayer MoS_2 FET device measured by switching source and drain electrodes (between electrode 1 and 2). Inset in **c** is the optical image of the AB stacking bilayer MoS_2 FET device.

We also performed electrical studies on another AB stacking bilayer FET device with the similar rectification characteristic and output curves (I_{ds}-V_{ds}) as those presented in Figure 5d and Supplementary Figures 14b, 14d and 14f. The output characteristics were measured by switching the source and drain electrodes (electrode 1 and 2) and the results are shown in Supplementary Figure 16c. It is clear that both output curves are of rectification characteristic

with slight inconsistency, meaning that both electrode contacts are slightly asymmetrical and of Schottky type. Similar effects on MoS_2 bilayer FET devices have been systematically studied by Bartolomeo et al.[28]. As they proved, such two asymmetrical Schottky contacts constitute two back-to-back junctions in the Electrode 1/MoS_2 channel/Electrode 2 configuration, which is thus responsible for the rectifying phenomena of MoS_2 bilayer FET devices including AB stacking samples in this work.

Supplementary Note VI. Characterization of WS_2 bilayer crystals

The representative optical images of WS_2 bilayer crystals are presented in Supplementary Figures 17a-17b, clearly reflecting the uniform optical contrast of the as-grown bilayer crystals. The representative AFM image in Supplementary Figure 17c exhibit a clean and smooth surface with a height profile of 1.5 nm across between the bilayer surface and the substrate surface, indicating the bilayer characteristic[35, 36]. The Raman and PL intensity mappings shown in Supplementary Figures 17d-17g all suggest a uniform surface of the WS_2 bilayer crystals. Supplementary Figures 17h and 17i compare Raman and PL spectra between the as-grown WS_2 bilayer crystals and CVD-grown WS_2 monolayer crystals, respectively. It is clear that the wavenumber difference between E^1_{2g} and A_{1g} of the WS_2 bilayer crystals is larger than that of the monolayer ones. Similar to the case of MoS_2 films, the PL intensity of the bilayer WS_2 crystals is greatly suppressed comparing with that of the monolayer ones due to the transition from a direct bandgap of monolayer to an indirect bandgap of bilayer[37]. Furthermore, the dominant PL peak has a redshift and at the same time there is a new PL peak emerging at about 711 nm, which can be ascribed to the band splitting caused by spin orbit coupling in bilayer WS_2[37, 38].

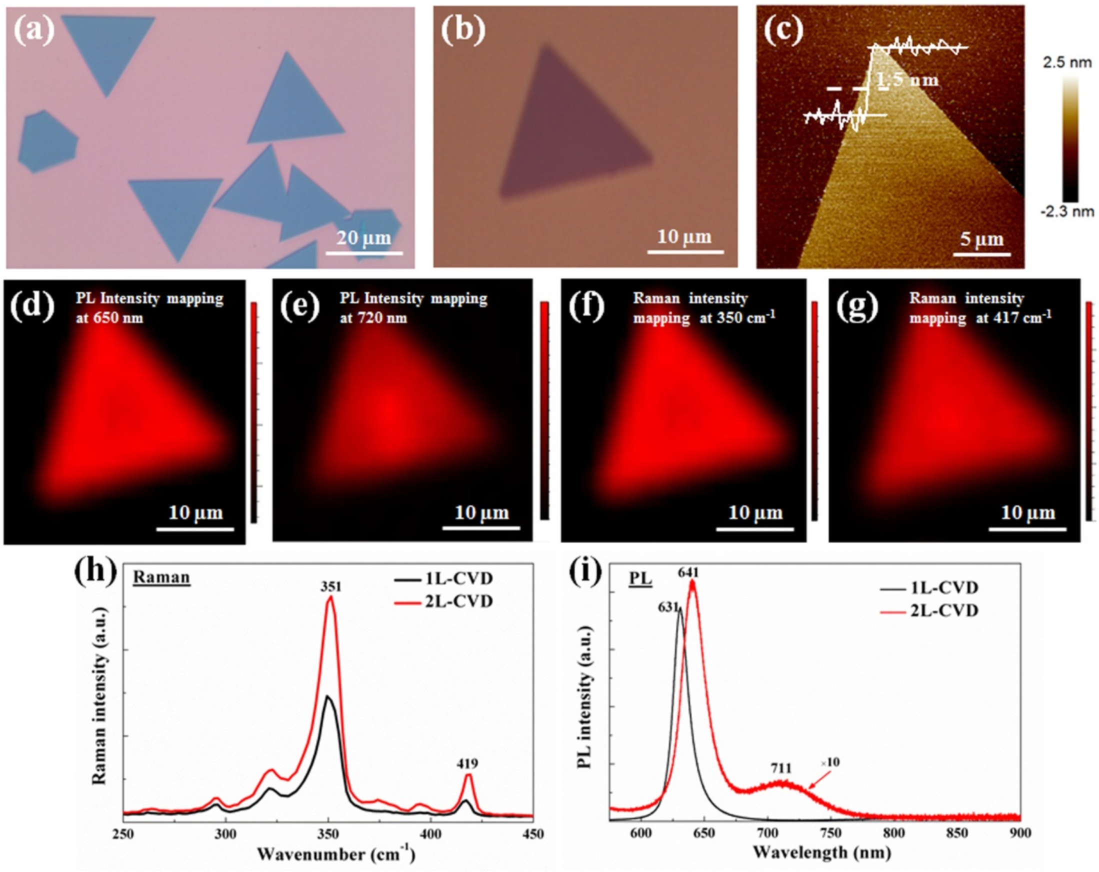

Supplementary Figure 17. The representative optical images **a-b** and AFM surface morphology **c** of the as-grown WS_2 bilayer crystals; the PL intensity mappings at 650 nm **d** and 720 nm **e** as well as the Raman intensity mappings at E_{2g}^1 (350 cm^{-1}) **f** and A_{1g} (417 cm^{-1}) **g** of the as-grown WS_2 bilayer crystals; Raman **h** and PL **i** spectra of the as-grown WS_2 bilayer crystals and CVD-grown WS_2 monolayer crystals.

Supplementary Note VII. Composition control in TMD bilayer alloys

The composition of ternary alloy $Mo_{1-x}W_xS_2$ and quaternary alloy $Mo_{1-x}W_xS_{2(1-y)}Se_{2y}$ can be tuned by adjusting the ratio of these source powders. As a proof of concept, we just show some preliminary results on quaternary alloy $Mo_{1-x}W_xS_{2(1-y)}Se_{2y}$ in Supplementary Figure 18. By fixing MoO_3, S and Se powder amounts and varying WO_3 powder amount, the values of x and y can be well controlled. Raman and PL spectroscopies are powerful and nondestructive characterization tools to determine the kinds of TMD materials[39,40] and their elemental composition[3,41]. As can be seen from Supplementary Figure 18a, with the amount of WO_3

increasing, the WS_2-related Raman peaks become more and more obvious and have a little redshift. At the same time, the MoS_2-related E^1_{2g} modes are gradually red-shifted while the MoS_2-related A_{1g} modes are gradually blue-shifted. On the other hand, as shown in Supplementary Figure 18b, with the amount of WO_3 increasing, the three PL peaks of the quarternary bilayer crystals all have some red shift. Both the Raman and PL results verify that the value of x and y in $Mo_{1-x}W_xS_{2(1-y)}Se_{2y}$ can be controlled by our reverse-flow chemical vapour epitaxy method.

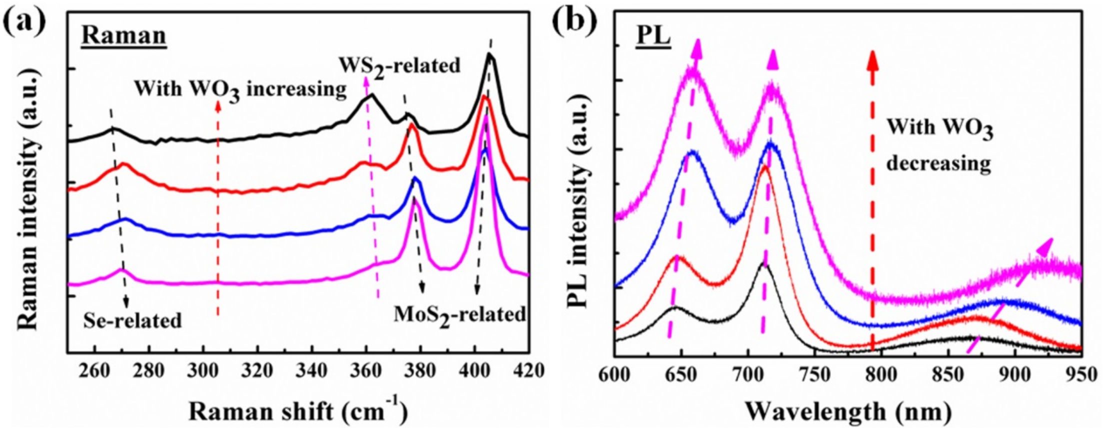

Supplementary Figure 18. **a** Raman and **b** PL spectra of the $Mo_{1-x}W_xS_{2(1-y)}Se_{2y}$ bilayer alloys obtained by fixing MoO_3, S and Se powder amounts and varying WO_3 powder amount using our reverse-flow chemical vapour epitaxy method.

Supplementary References

1. Lee, Y. H. et al. Synthesis of large-area MoS_2 atomic layers with chemical vapor deposition. *Adv. Mater.* **24**, 2320-2325 (2012).

2. Zhang, X. et al. Shape-uniform, High-quality Monolayered MoS_2 crystals for gate-tuneable photoluminescence. *ACS Appl. Mater. Inter.* **9**, 42121–42130 (2017).

3. Yan, J. et al. Stacking-dependent interlayer coupling in trilayer MoS_2 with broken inversion symmetry. *Nano Lett.* **15**, 8155-8161 (2015).

4. Xia, M. et al. Spectroscopic signatures of AA' and AB stacking of chemical vapor deposited bilayer MoS_2. *ACS Nano* **9**, 12246-12254 (2015).

5. Yan, A. et al. Identifying different stacking sequences in few-layer CVD-grown MoS_2 by

low-energy atomic-resolution scanning transmission electron microscopy. *Phys. Rev. B* **93**, 184–189 (2016).

6. Gong, Y. et al. Synthesis of millimeter-scale transition metal dichalcogenides single crystals. *Adv. Funct. Mater.* **26**, 2009-2015 (2016).

7. Cao, D., Shen, T., Liang, P., Chen, X. S. & Shu, H. B. Role of chemical potential in flake shape and edge properties of monolayer MoS_2. *J. Phys. Chem. C* **119**, 4294-4301 (2015).

8. Schweiger, H., Raybaud, P., Kresse, G. & Toulhoat, H. Shape and edge sites modification of MoS_2 catalytic nanoparticles induced by working conditions: A theory study. *J. Catal.* **207**, 76-87 (2002).

9. Wang, S. S., Sawada, H., Allen, C. S., Kirkland, A. I. & Warner, J. H. Orientation dependent interlayer stacking structure in bilayer MoS_2 domains. *Nanoscale* **9**, 13060-13068 (2017).

10. Yang, S. X., Kang, J., Yue, Q. & Yao, K. Vapor phase growth and imaging stacking order of bilayer molybdenum disulfide. *J. Phys. Chem. C* **118**, 9203-9208 (2014).

11. Annamalai, M. et al. Surface energy and wettability of van der Waals structures. *Nanoscale* **8**, 5764-5770 (2016).

12. Yoo, Y. D.，Degregorio, Z. P. & Johns, J. E. Seed crystal homogeneity controls lateral and vertical heteroepitaxy of monolayer MoS_2 and WS_2. *J. Am. Chem. Soc.* **137**, 14281-14287 (2015).

13. He, J. G., Hummer, K. & Franchini, C. Stacking effects on the electronic and optical properties of bilayer transition metal dichalcogenides MoS_2, $MoSe_2$, WS_2, and WSe_2. *Phys. Rev. B* **89**, 075409 (2014).

14. Yan, A. M. et al. Identifying different stacking sequences in few-layer CVD-grown MoS2 by low-energy atomic-resolution scanning transmission electron microscopy. *Phys. Rev. B* **93**, 041420 (2016).

15. Ni, Z. H. et al. Graphene thickness determination using reflection and contrast spectroscopy. *Nano Lett.* **7**, 2758-2763 (2007).

16. Grimme, S., Antony, J., Ehrlich, S. & Krieg, H. A consistent and accurate ab initio parametrization of density functional dispersion correction (DFT-D) for the 94 elements H-Pu. *J. Chem. Phys.* **132**, 154104 (2010).

17. Tawfik, S. A., Gould, T., Stampfl, C. & Ford, M. J. Evaluation of van der Waals density functionals for layered materials. *Phys. Rev. Mater.* **2**, 034005 (2018).

18. Bhattacharyya, S. & Singh, A. K. Semiconductor-metal transition in semiconducting bilayer sheets of transition-metal dichalcogenides. *Phys. Rev. B* **86**, 075454 (2012).

19. Xiao, J., Long, M. Q., Li, X. M., Zhang, Q. T., Xu, H. & Chan, K. S. Effects of van der Waals interaction and electric field on the electronic structure of bilayer MoS_2. *J. Phys.: Condens. Matter* **26**, 405302 (2014).

20. Xiao, D., Liu, G. B., Feng, W. X., Xu, X. D. & Yao, W. Coupled Spin and Valley Physics in Monolayers of MoS2 and Other Group-VI Dichalcogenides. *Phys. Rev. Lett.* **108**, 196802 (2012).

21. Zhu, Z. Y., Cheng, Y. C. & Schwingenschlogl, U. Giant spin-orbit-induced spin splitting in two-dimensional transition-metal dichalcogenide semiconductors. *Phys. Rev. B* **84**, 153402 (2011).

22. Kan, M. et al. Structures and phase transition of a MoS_2 monolayer. *J. Phys. Chem. C* **118**, 1515-1522 (2014).

23. Mak, K. F., Lee, C., Hone, J., Shan, J. & Heinz, T. F. Atomically Thin MoS_2: A New Direct-Gap Semiconductor. *Phys. Rev. Lett.* **105**, 136805 (2010).

24. Bronsema, K. D., de Boer, J. L. & Jellinek, F. On the structure of molybdenum diselenide and disulfide. *Z. Anorg. Allg. Chem.* **540**, 15-17 (1986).

25. Amani, M. et al. Electrical performance of monolayer MoS_2 field-effect transistors prepared by chemical vapor deposition. *Appl. Phys. Lett.* **102**, 193107 (2013).

26. Radisavljevic, B., Radenovic, A., Brivio, J., Giacometti, V. & Kis, A. Single-layer MoS_2 transistors. *Nat. Nanotechnol.* **6**, 147-150 (2011).

27. Xie, X. J. et al. Low-frequency noise in bilayer MoS_2 transistor. *ACS Nano* **8**, 5633-5640 (2014).

28. Bartolomeo, A. D. et al. Asymmetric schottky contacts in bilayer MoS_2 field effect transistors. *Adv. Funct. Mater.* **28**, 1800657 (2018).

29. Mcdonnell, S.，Addou, R.，Buie, C., Wallace, R. M. & Hinkle, C. L. Defect-dominated doping and contact resistance in MoS_2. *ACS Nano* **8**, 2880-2888 (2014).

30. Zhong, H. X. et al. Interfacial properties of monolayer and bilayer MoS_2 contacts with metals: beyond the energy band calculations. *Sci. Rep.* **6**, 21786 (2016).

31. Wang, H. et al. Integrated circuits based on bilayer MoS_2 transistors. *Nano Lett.* **12**,

4674-4680 (2012).

32. Lu, C. L., Chang, C. P., Huang, Y. C., Ho, J. H., Hwang, C. C. & Lin, M. F. Electronic properties of AA- and ABC-stacked few-layer graphites. *J. Phys. Soc. Jpn.* **76**, 024701 (2007).

33. Zhang, R. Q., Li, B. & Yang, J. L. Effects of stacking order, layer number and external electric field on electronic structures of few-layer C_2N-h2D. *Nanoscale* **7**, 14062-14070 (2015).

34. Charlier, J. C.，Gonze, X. & Michenaud, J. P. First-principles study of the stacking effect on the electronic properties of graphite(s). *Carbon* **32**, 289-299 (1994).

35. Cong, C. et al. Synthesis and optical properties of large-area single-crystalline 2D semiconductor WS_2 monolayer from chemical vapor deposition. *Adv. Opt. Mater.* **2**, 131-136 (2014).

36. Zhang, Y. et al. Controlled growth of high-quality monolayer WS_2 layers on sapphire and imaging its grain boundary. *ACS Nano* **7**, 8963-8971 (2013).

37. Jo, S., Ubrig, N., Berger, H., Kuzmenko, A. B. & Morpurgo, A. F. Mono- and bilayer WS_2 light-emitting transistors. *Nano Lett.* **14**, 2019-2025 (2014).

38. Nayak, P. K., Lin, F. C., Yeh, C. H., Huang, J. S. & Chiu, P. W. Robust room temperature valley polarization in monolayer and bilayer WS_2. *Nanoscale* **8**, 6035-6042 (2016).

39. Mitioglu, A. A. et al. Optical manipulation of the exciton charge state in single-layer tungsten disulfide. *Phys. Rev. B* **88**, 245403 (2013).

40. Chiu, M. H. et al. Spectroscopic signatures for interlayer coupling in MoS_2-WSe_2 van der Waals Stacking. *ACS Nano* **8**, 9649 (2014).

41. Wang, X. H. et al. Photoluminescence and Raman mapping characterization of WS_2 monolayers prepared using top-down and bottom-up methods. *J. Mater. Chem. C* **3**, 2589-2592 (2015).
